# PRO-NOVELTY: Patient-Reported Outcomes in NOse VEstibule interventionaL radioTherapY (brachytherapy)

**DOI:** 10.3390/jcm13164683

**Published:** 2024-08-09

**Authors:** Luca Tagliaferri, Elisabetta Sciurti, Bruno Fionda, Antonella Loperfido, Valentina Lancellotta, Elisa Placidi, Claudio Parrilla, Maria Concetta La Milia, Enrico Rosa, Mario Rigante, Martina De Angeli, Patrizia Cornacchione, Jacopo Galli, Francesco Bussu, Maria Antonietta Gambacorta

**Affiliations:** 1 UOC Degenze di Radioterapia Oncologica, Dipartimento di Diagnostica per Immagini e Radioterapia Oncologica, Fondazione Policlinico Universitario A. Gemelli IRCCS, 00168 Rome, Italy; 2 Istituto di Radiologia, Università Cattolica del Sacro Cuore, 00168 Rome, Italy; 3 Otolaryngology Unit, San Camillo Forlanini Hospital, 00152 Rome, Italy; 4 UOC Fisica per le Scienze della Vita, Dipartimento di Diagnostica per Immagini e Radioterapia Oncologica, Fondazione Policlinico Universitario A. Gemelli IRCCS, 00168 Rome, Italy; 5 Unità Operativa Complessa di Otorinolaringoiatria, Dipartimento di Scienze dell’Invecchiamento Neurologiche Ortopediche e del Testa-Collo, Fondazione Policlinico Universitario A. Gemelli IRCCS, 00168 Rome, Italy; 6 eCampus University, 22060 Novedrate, Italy; 7 Sezione di Otorinolaringoiatria, Dipartimento Universitario Testa-Collo e Organi di senso, Università Cattolica del Sacro Cuore, 00168 Rome, Italy; 8 Otorhinolaryngology Division, Azienda Ospedaliero Universitaria Sassari, 07100 Sassari, Italy; 9 Department of Medicine, Surgery and Pharmacy, University of Sassari, 07100 Sassari, Italy; 10 UOC Servizi di Radioterapia Oncologica, Dipartimento di Diagnostica per Immagini e Radioterapia Oncologica, Fondazione Policlinico Universitario A. Gemelli IRCCS, 00168 Rome, Italy

**Keywords:** nose vestibule, nasal vestibule, interventional radiotherapy, brachytherapy, quality of life

## Abstract

**Background:** The aim of this paper is to evaluate the impact on the quality of life of the treatment of nasal vestibule tumors by interventional radiotherapy (IRT-brachytherapy) through a patient reported outcome questionnaire. **Methods:** We prospectively collected data about patients undergoing IRT according to our institutional schedule of 44 Gy delivered in 14 fractions twice a day. We recorded both acute toxicity data, using the Common Terminology Criteria for Adverse Events (CTCAE) version 5.0, and quality of life data, using the 22-item Sino-Nasal Outcome Test (SNOT-22) at baseline (T0), at 1 month (T1), at 3 months (T3), and at 6 months (T6). **Results:** We enrolled 10 consecutive patients treated between February 2023 and October 2023. The decrease in terms of SNOT-22 mean value was statistically significant from T0 and T6 with a *p*-value < 0.001. A noteworthy clinical finding is that quality of life improved regardless of the occurrence of G1-G2 side effects. **Conclusions:** Using SNOT-22 on patients with nasal vestibule carcinoma treated with IRT has shown an improvement in quality of life that is not strictly dependent on the occurrence of expected G1-G2 side effects.

## 1. Introduction

Nasal vestibule (NV) tumors have traditionally been regarded as an uncommon disease accounting for less than 1% of all head and neck malignancies [1]. A recent nationwide study performed in the Netherlands revealed that the incidence of NV tumors ranges from 0.24 to 0.47 per 100,000 people [2]. In a similar study conducted in Denmark, the estimated annual incidence of NV tumors was found to be 0.32 per 100,000 people; however, the authors pointed out the risk of underestimation, which could be related, in part, to underreporting since some tumors could be classified as skin malignancies [3,4].

Historically, the first evidence regarding the use of radiotherapy for the treatment of NV carcinoma dates back to 1974, when Haynes et al. reported nine cases treated by interventional radiotherapy (IRT-brachytherapy) [5].

In 2024, for the first time, the oncological committee of the Italian society of otorhinolaryngology released specific recommendations identifying IRT as the standard of care for NV tumors; in fact, the authors underline that IRT provides local control comparable to surgery, but it may allow organ preservation and, consequently, better function and aesthetic appearance [6]. It should be underlined that even though IRT is a valid therapeutic tool for NV, it has some limitations: in fact, in very advanced cases, especially those with bone involvement, it should be carefully discussed within the frame of a multidisciplinary approach.

However, currently, there are only retrospective studies on the functional outcomes and side effects of IRT in the management of NV malignancies and no specific data on quality of life (QoL) [7,8].

In particular, our preliminary evidence comparing the functional outcomes of surgery versus IRT suggests that the latter provides better results [9].

In the clinical scenario of head and neck cancer patients, there are several patient-reported outcome (PRO) questionnaires available. However, most of them do not include specific questions about symptoms regarding the midface or nose, and none is specific for NV [10,11].

A commonly used PRO questionnaire in rhinology is the 22-item Sino-Nasal Outcome Test (SNOT-22), which detects the severity of symptoms (nasal, facial, and otologic), the social and emotional impact of the nasal disease and its consequences on sleep and productivity [12]. This test has been translated and validated in many world languages including Italian [13]. 

Noteworthy is the fact that, very recently, several authors have started to use the SNOT-22 in the oncological setting and specifically for sinonasal malignancies [14,15,16,17]. 

The aim of this paper is to evaluate the impact on the quality of life of the treatment of NV tumors by IRT through a PRO questionnaire, namely, the SNOT-22. 

## 2. Materials and Methods

We prospectively collected data about patients using the institutional electronic database fully complying with General Data Protection Regulations (GDPR). 

After a multidisciplinary evaluation considering function, cosmesis, and patient preference, the decision to employ IRT rather than a surgical approach was made with the aim of attaining optimal overall outcomes. All patients were treated at our institutional interventional oncology center (IOC) following the principle of anatomical implantation (with additional endocavitary implant in selected cases); the overall delivered dose was 44 Gy in 14 fractions twice a day. Further details about schedule and implant techniques have been published in our previous paper [18].

Each patient underwent an extensive physical examination, followed by a biopsy of the primary lesion to obtain samples for histological analysis. In addition, either head-and-neck computed tomography (CT) or magnetic resonance imaging (MRI) was performed to accurately determine the disease stage. Patients were required to give informed consent for both the medical procedures and subsequent treatment. They also had to consent to the use of their personal data for scientific research purposes.

Toxicities were objectively evaluated based on the Common Terminology Criteria for Adverse Events (CTCAE) version 5.0 at baseline, at 1 month, at 3 months, and at 6 months; in more detail, we focused on items related to the upper airways including rhinorrhea, nasal congestion, postnasal drip, sinus disorder, and sinus pain [19].

The subjective evaluation of NV squamous cell carcinoma (SCC) patients treated through IRT was performed using SNOT-22 following the same timing of the objective evaluation.

The SNOT-22 represents a PRO questionnaire composed of twenty-two personalized questions concerning health-related QoL and symptom severity in patients with nasal diseases. The outcomes assessed are classified into two groups: physical symptoms (items 1–12), which include symptoms related to the nasal district (items 1–8), ear symptoms (items 9–11), and facial symptoms (item 12); and QoL and state (items 13–22) which specifically assess patients’ sleep (items 13–16) and mental sensations (items 17–22). The total result score can vary from 0 to 110, and higher scores mean worse symptoms. Specifically, the sum of the scores of each SNOT-22 question is equal to the severity of nasal symptoms: a score greater than 50 corresponds to severe symptoms, between 20 and 49 to moderate symptoms, and less than 20 to mild symptoms. 

We utilized Excel data analysis tools to compute the mean values, and statistical significance was evaluated using a single-tailed paired *t*-test.

## 3. Results

Overall, we enrolled 10 patients in this observational prospective study treated between February 2023 and October 2023. A detailed list of the patients’ features is reported in Table 1.

Regarding objective symptoms, rhinorrhea, defined as a disorder characterized by excessive mucous secretions draining from the nose, was present in all patients after the treatment at the first evaluation (1 month after IRT–T1) and subsequently progressively declined. 

Closely related to rhinorrhea was nasal congestion, defined as the obstruction of the nasal passage due to mucosal edema, which was present in all patients at T1 requiring medical intervention with topical instillation (G2) and then remained in 50% of patients at T6 without the need for medical intervention (G1). 

Interestingly, postnasal drip, defined as a disorder characterized by excessive mucous secretion in the back of the nasal cavity or throat, causing sore throat and/or coughing, was present only in 20% of the patients at T1.

On the other hand, sinus disorder, defined as an involvement of the paranasal sinuses, and sinus pain, defined as a sensation of marked discomfort in the face, between the eyes, or upper teeth originating from the sinuses, were not found in any of the patients analyzed.

A comprehensive summary of all objective symptoms is presented in Table 2.

**Figure 1:**
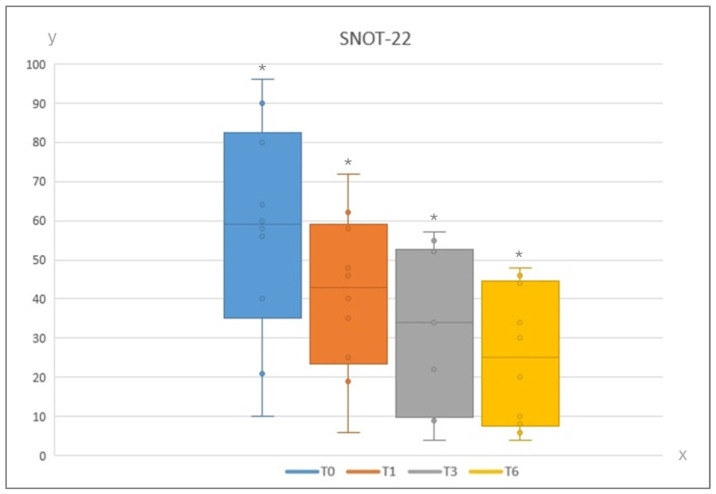
SNOT-22 values at different time intervals (on the x axis timing of subjective reporting are reported; on y axis the actual values of SNOT-22 are reported). Legend. SNOT-22: 22-item Sino-Nasal Outcome Test; T0: baseline; T1: at 1 month after IRT; T3: at 3 months after IRT; T6: at 6 months after IRT. *—statistically significat (*p* < 0.05).

The mean SNOT-22 value was 58 at baseline, 41 at 1 month, 31 at 3 months and 25 at 6 months, as reported in Figure 1.

The decrease in terms of SNOT-22 mean value was statistically significant from T0 and T6 with a *p*-value < 0.001. However, when analyzing the single time intervals, we found that the trend to decrease turned out to be statistically significant in all cases: T0–T1 had a *p*-value of <0.015, T1–T3 had a *p*-value of <0.002, and T3–T6 had a *p*-value of <0.005.

**Figure 2:**
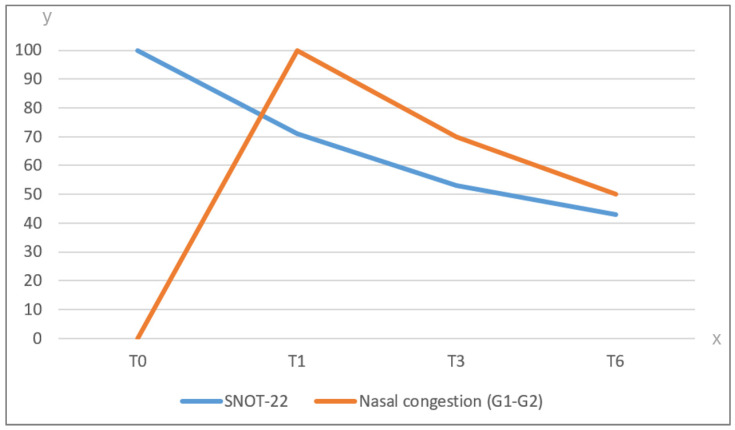
Comparison between trend of PROs and side effects after IRT (on the x axis timing of objective and subjective reporting are reported; on the y axis the percentage of side effects and PROs are reported). Legend. SNOT-22: 22-item Sino-Nasal Outcome Test; T0: baseline; T1: at 1 month after IRT; T3: at 3 months after IRT; T6: at 6 months after IRT; G1: grade 1: G2: grade 2; Nasal congestion (G1–G2): sum of G1 and G2 adverse events.

A point of significant clinical relevance is the observation that, when comparing the trends of patient-reported outcomes (PROs) and side effects, the quality of life improved independently from the occurrence of side effects, as graphically shown in Figure 2.

With regard to local control, no recurrence was observed by clinical examination and imaging, and the overall patient satisfaction regarding the aesthetic result was very good since no nasal deformities were detected.

## 4. Discussion

To the very best of our knowledge, this is the first study in the literature prospectively evaluating the QoL in patients affected by NV SCC and treated with exclusive IRT, the new standard treatment for this, until now, underestimated and potentially devastating disease.

NV stands out prominently within the body due to its central position on the face, making even minor deformities and scars from surgical procedures highly noticeable and socially impactful. Moreover, NV SCC tends to invade the nasal cartilages, necessitating extensive surgical removal. Consequently, reconstructive surgery becomes exceedingly challenging, since faithfully replicating the intricate contours and hollows shaped by the nasal cartilages and bones is virtually impossible [20].

From an anatomical perspective, NV is situated at the foremost section of the nasal cavity, comprising lateral, medial, and inferior walls supported by nasal cartilages. Its lining is skin to skin, featuring a keratinized stratified squamous epithelium; this structure allows NV SCC to extend directly to the skin, often leading to misdiagnosis as skin cancer [21]. It is worth noting that, according to certain authors, the incidence of NV may be underestimated due to misclassification and misdiagnosis. This is compounded by the absence of clearly defined radiological boundaries for the NV, resulting in a lack of specific WHO topographic codes in cancer registries. To address this issue, some have proposed using a tangent plane at the pyriform opening as the posterior limit of the NV, potentially enabling the creation of a specific topographic code for identification purposes.

Various systems exist for categorizing NV SCC [22], with the most widely utilized and recognized being the Wang system introduced in 1976 [23]. However, newer classification systems have recently emerged, showing improved correlation with prognosis [24].

Managing NV SCC poses a dual challenge for physicians in terms of both oncological and functional considerations. Currently, surgical intervention, external beam radiotherapy (EBRT), and IRT stand as the available treatment modalities in clinical practice [25]. Under an oncological perspective, interstitial IRT has been demonstrated as an effective oncological treatment for primary NV SCCs, showing a control rate comparable to surgical intervention, but with superior oncological and functional outcomes in managing NV SCC compared to EBRT [7,26].

Regarding aesthetic outcomes, there are reports highlighting that IRT is associated with good results in overall appearance [8] and that such results are in line with patients’ satisfaction [27]. It is noteworthy that the nasal cartilage tends to withstand radiation quite well when the trophic support of the perichondrium remains intact [28]. This is probably the main reason why patients treated with interstitial IRT showed significantly higher satisfaction with aesthetic outcomes than those undergoing surgery [29]. EBRT has been considered a viable alternative from an aesthetic perspective [30]. However, it has been demonstrated inferior under an oncological point of view [24]. IRT, besides offering superior local control, appears unaffected by the classical irradiation acute and late toxicities. In fact, nasal functions are affected by irradiation [31], and many disorders, such as crusting [32], nasal dryness and obstruction [33], dysgeusia, and dysosmia [34] in patients receiving previous irradiation of the nasal district are related to the alteration of physiological mechanisms by the mucosal toxicity of radiation therapy [35,36].

Contrarily, patients treated with IRT maintain a significantly preserved nasal physiology and cytological appearance of the nasal mucosa when compared to non-irradiated healthy individuals. Some authors attribute this functional preservation to the swift decline in IRT dosage, resulting in a substantial decrease in the irradiated mucosal area within the nasal and paranasal cavities.

As a result of all the above considerations, IRT is now regarded as a valid therapeutic option for the treatment of early-stage primary malignancies in the nasal vestibule.

The most frequently reported side effects of combined mold-based intracavitary and interstitial pulsed-dose-rate (PDR) brachytherapy include nasal crusting (66.1%), ulcers (16.1%), chondroradionecrosis (9.7%), and epistaxis (6.5%). Notably, chondroradionecrosis occurs much less frequently (0–4%) with the interstitial high-dose rate (HDR) approach. This suggests that image guidance, accumulated experience over time, and new anatomical implantation techniques for NV carcinomas contribute to better outcomes [18,37].

A significant late complication observed in clinical trials involving IRT for NV SCC treatment is chondronecrosis, often associated with nasal septum and/or alar perforations. Studies indicate that this particular adverse event occurs more frequently with interstitial implants compared to endocavitary and/or mold approaches. In our opinion, chondronecrosis results from IRT-related perichondrial rupture, disrupting the connective tissue layer that nourishes cartilage [37]. To preserve perichondrial and cartilage anatomy during IRT, some suggest using plastic tubes implanted along subperichondral planes, tailoring the NV implant geometry to each patient’s nasal anatomy rather than relying on the “classical” Paris system rules [38]. Additionally, a combination of intracavitary and interstitial implants may be considered, especially for malignancies extending posteriorly into the nasal fossa, potentially benefiting from additional dose modulation [39]. All of these new rules form the novel concept of “anatomical implantation” [24].

The use of IRT offers several notable advantages, including straightforward access to the clinical target volume; ease of tube placement even under local anesthesia, which is particularly beneficial for elderly or frail patients; the absence of critical structures nearby that need preservation; and the ability to deliver very high doses or provide a dose boost.

Despite these benefits, managing toxicity remains a critical aspect of treatment planning for curative radiotherapy doses. Although vital structures such as the central nervous system and carotid axis, which are significant concerns in head and neck radiation oncology, are typically distant from the clinical target volume (CTV), the eyes can be at risk in cases where lesions have extended cranially. The administration of very high doses, combined with the intrinsic dose inhomogeneity characteristic of IRT, can lead to considerable toxicities affecting soft tissues, skin, and mucosal surfaces. Therefore, implementing strategies to reduce the dose within the treatment volume and to protect nearby tissues and organs at risk is essential.

When considering the treatment volume, there are two main factors to address. The first is the technique used for implantation, which can be either interstitial or endocavitary, utilizing nasal packing or customized molds for stabilization. In more complex cases, a combination of approaches, including endocavitary, interstitial, and/or contact methods (as used in skin cancer treatments), can be employed. Interstitial implantation can follow the established Paris system or newer anatomical implantation methods. The second factor involves the distribution of the dose within the implant, with the dose non-uniformity ratio (DNR) serving as a common parameter to assess the volume receiving 1.5 times the prescribed dose.

To mitigate the dose exposure to organs at risk, two different approaches have been proposed by some researchers. The first involves using protective shields for fixed organs, such as the eyes, where metal shields can be utilized to decrease the dose received by the lens. The second approach includes the use of a customized mouth swab to move mobile organs away from the radiation field. For example, a mouth swab can be used to displace anatomical structures such as the oral cavity, buccal mucosa, lower lip, and mandible, thereby reducing the dose absorbed by these areas [40].

In the existing literature, aside from investigations into the oncological, functional, and aesthetic outcomes as well as the side effects of IRT in managing NV SCC, there is a lack of specific data regarding the QoL of these patients. The limited clinical experiences documented in the literature relate to studies assessing health-related QoL in patients’ post-radical resection of nasal skin cancer, utilizing rhinoplasty-specific questionnaires [41,42,43].

More generally, head and neck cancer, encompassing malignancies in areas such as the oral cavity, pharynx, larynx, nasal cavity, and salivary glands, presents a significant clinical challenge due to its complex anatomy and critical functional regions involved in breathing, eating, speaking, and sensory perception. Consequently, in real life, head and neck cancer patients experience major changes in their appearance, speech, eating, work efficiency, and, more generally, in their daily routines. This results in a high impact on QoL and all aspects of patients’ life (not only physical, but also emotional, social, cognitive and role functioning) [44].

According to the World Health Organization (WHO), QoL represents “an individual’s perception of their position in life in the context of the culture and value systems in which they live and in relation to their goals, expectations, standards, and concerns” [45]. Therefore, this term refers to a subjective construct that denotes how a patient perceives his or her health and well-being, as QoL encompasses a wide range of different aspects, including physical health, emotional state, social relationships, level of independence, personal beliefs, interaction with family, and the cultural environment in which a person lives. Emotionally, the burden of dealing with head and neck cancer and its treatment can be overwhelming. The drastic changes in appearance, such as swelling, redness, and, in some cases, disfigurement due to surgical removal of the tumor or the possible side effects of chemotherapy and/or radiation therapy, can have a major impact on a patient’s self-esteem and body image. Many patients suffer from anxiety and depression because of fears about their prognosis, the stigma associated with the visible signs of the disease, and the frustration of having to deal with ongoing physical discomfort. The psychological impact is compounded by the often prolonged and intensive nature of adjuvant treatments, which require frequent hospital visits and interrupt normal daily routines. This interruption can lead to feelings of isolation, especially if patients are unable to engage in social or work activities due to fatigue and other side effects. According to some authors, these emotional disturbances commonly occur immediately after cancer diagnosis and in the initial phase of treatment in which patients go through a period of adaptation to the disease that lasts from weeks to months. Specifically, eating disorders, salivary dysfunction, and problems in social contacts have been reported to be the main risk factors for depression in patients with head and neck cancer [46].

Interestingly, the clinical and demographic characteristics that most impact the QoL of head and neck cancer patients have been described in the literature. Among clinical predictors, the presence of a feeding tube is considered a strong negative predictor of QoL, but also multiple comorbid medical conditions, the presence of a tracheotomy tube, prior chemotherapy treatment, laryngectomy status, history of previous neck dissection, and radiation therapy (in descending severity order) demonstrated strong negative effects on several QoL scales. It is therefore of paramount importance that physicians consider the impact of clinical treatment modalities on QoL when discussing treatment options for these patients [47].

The instruments commonly used in clinical practice to measure health-related QoL are patient-reported outcome (PRO) questionnaires. The term PRO refers to a health outcome reported directly by the patient who experienced it. It is contrasted with an outcome reported by others, such as an outcome reported by a physician or an outcome reported by a nurse. This is because, in recent decades, healthcare systems have increasingly recognized patients’ perspectives as being of primary importance in ensuring that services are of high quality and delivered fairly and safely [48]. The increasing use of PRO measures has been part of this shift [49,50]. The most frequently used PRO questionnaires evaluate one of the following domains: functioning (disability), symptoms (impairments) and other aspects of well-being, health status, QoL, health-related QoL, general health perceptions, and healthcare reports and ratings. Measures of symptoms generally focus on a range of impairments or on a specific impairment such as pain, anxiety, or depression. Contrarily, measures of functioning assess specific activities such as locomotor activities, personal care, and activities of daily living. Health-related QoL tools are usually represented by multi-dimensional questionnaires assessing a combination of aspects of disability and/or impairments and describe a patient’s health status. In contrast, QoL questionnaires go beyond disability and impairment by asking about the patient’s capacity to fulfill their needs and also about their psychological and emotional response to their restrictions [51].

Among PRO questionnaires, the SNOT-22 stands out as one of the most utilized tools in rhinology for assessing patients’ QoL. Widely recognized as a valid PRO questionnaire, SNOT-22 is extensively recommended in clinical practice, particularly for benign sinonasal conditions. In particular, SNOT represents a PRO questionnaire largely used in clinical practice as it covers a wide range of health and health-related QoL aspects, such as physical problems, associated functional limitations, and, not least, the emotional and psychological consequences of illness [52].

More specifically, regarding questionnaire structure and its twenty-two questions, the SNOT-22 assesses five different basic domains: three sinus-specific symptom domains (rhinologic symptoms such as blockage/congestion of nose and postnasal discharge, extranasal rhinologic symptoms such as taste/smell impairment, and auricular/facial symptoms such as ear fullness and facial pain/pressure) and two domains concerning general health-related QoL (impact on the patient’s psychological aspect and sleep function) [53].

SNOT-22 was first used by the Royal College of Surgeons of England in a large multicenter study about sinonasal surgery, which began in 2000. Since the results of the study were reported in the literature [54], SNOT-22 gained wide popularity. Indeed, the SNOT questionnaire has been applied in multiple studies on chronic rhinosinusitis (CRS) [55], as well as in an increasingly wide range of rhinologic and non-rhinologic surgical procedures and clinical conditions, including sinus surgery [56], septorhinoplasty [57], septoplasty [58], rhinitis [59], hereditary hemorrhagic telangiectasia (HHT) [60], antineutrophil cytoplasmic antibody (ANCA)-associated vasculitis [61], obstructive sleep apnea syndrome (OSAS), radiofrequency volumetric tissue reduction of inferior turbinates (RVTR) [62], asthma, and chronic obstructive pulmonary disease (COPD) [63]. 

The widespread use of SNOT-22 is because this PRO measure has proven to be a valid and easy-to-use tool in routine clinical practice, both for assessing the impact of the disease on the patient’s QoL and for measuring the outcome of surgery.

Interestingly, this questionnaire has also found significant application in oncology, particularly concerning the sinonasal region and EBRT.

Chow et al. [11] conducted a review to assess how sinonasal tumors and their treatment impact patient QoL using PRO questionnaires. They discovered that several factors were linked to a poorer QoL, such as age over 60, smoking history, advanced disease stage, a timeframe of less than six months post-surgery, previous and additional treatments like chemotherapy and radiation, and a malignant type of tumor. The authors concluded that there is not a universally accepted PRO questionnaire for both benign and malignant nasal sinus tumors. They noted that while patients with benign lesions generally return to their pre-treatment QoL, this is not the case for those with sinonasal tumors, who typically experience a lower QoL compared to before treatment.

Fleseriu et al. [17] utilized the questionnaire to pinpoint factors affecting QoL before treatment in patients with sinonasal tumors. Their goal was to customize multidisciplinary counseling and management strategies. The authors conducted a multicenter observational study, enrolling 204 previously untreated patients and gathering baseline QoL questionnaires, demographic details, comorbidities, histopathology, tumor extent, and symptoms. Within the rhinologic subdomain, women, patients with industrial exposure, and those experiencing epistaxis showed worse outcomes.

Maoz et al. [15] carried out a prospective, multisite, and longitudinal observational study to analyze the QoL of 194 patients with sinonasal conditions undergoing definitive treatment for curative purposes. Their findings indicated that all QoL scores were diminished at the start, but improved throughout treatment, with particularly noticeable enhancements in SNOT-22 indices within three months.

Grimm et al. [16] conducted a prospective multicenter study involving 234 patients diagnosed with sinonasal tumors. They assessed the patients’ overall sinonasal QoL using the SNOT-22 questionnaire. SNOT-22 scores were gathered at diagnosis and during post-treatment follow-ups of up to five years (with an average follow-up period of 22 months). The study revealed significant improvements in the rhinologic, psychological, and sleep-related aspects of QoL compared to baseline. When comparing various treatment methods, the authors noted that patients who underwent additional postoperative radiotherapy, either alone or in combination, experienced more pronounced issues with rhinologic and sleep-related disorders.

Our study has several limitations, and the first one is the fact that ours is a single-center experience. 

Another important limitation is the fact that the follow-up period is only of six months, and this might not be long enough to detect major changes such as anatomical changes and/or shrinking of the nose. 

Another limitation of this study is represented by the fact that the cohort of patients is very small; even though the cohort presented in this study is limited, it still represents a valid series considering the rarity of the disease, the prospective nature of this report, and the short accrual time of less than one year.

Multicentric studies with larger numbers and longer follow-up times are, therefore, desirable to investigate the topic of QoL in NV patients treated by IRT. Moreover, there is a need to foster the use of PROs in clinical practice, and it would be desirable to investigate other questionnaires (e.g., Utrecht/SNOT-23/Rhino).

## 5. Conclusions

PROs may play a crucial role in evaluating the quality of life and long-term satisfaction of oncological patients undergoing interventional radiotherapy (brachytherapy). Our prospective observational series using SNOT-22 on patients with NV carcinoma treated by IRT is a preliminary experience reporting an improved quality of life in this clinical setting.

## Figures and Tables

**Table 1:** Patients’ characteristics.

Main Features	Outcomes
n. of patients	10
M/F	50%/50%
Median age	70 years (range 45–81)
Histology	SCC 100%
Wall of origin	Lateral 60% Inferior 40%
Wang staging system	T1 30% T2 70%
Rome staging system	T1 20% T2a 30% T2b 40% T3 10%
N status	Negative (100%)
M status	Negative (100%)

Legend. M: male; F: female; SCC: squamous cell carcinoma; N: lymph node; M: distant metastases.

**Table 2:** Side effects at 1, 3, and 6 months post IRT.

Side Effects	At 1 Month	At 3 Months	At 6 Months
Rhinorrhea *	100%	70%	50%
Nasal congestion	G2 (100%)	G2 (50%)–G1 (20%)	G1 50%
Postnasal drip	G1 (20%)	-	-
Sinus disorder	-	-	-
Sinus Pain	-	-	-

* No grading is provided in accordance with Common Terminology Criteria for Adverse Events (CTCAE) v 5.0.

## Data Availability

The data presented in this study are available on request from the corresponding author due to privacy.
